# Gender differences in non-alcoholic fatty liver disease in obese children and adolescents: a large cross-sectional study

**DOI:** 10.1007/s12072-023-10596-9

**Published:** 2023-10-20

**Authors:** Binghan Jin, Zhaoyuan Wu, Shan Wang, Zhu Yu, Rahim Ullah, Xinyi Liang, Wei Wu, Ke Huang, Yan Ni, Jianbing Wang, Guanping Dong, Junfen Fu

**Affiliations:** 1 Department of Endocrinology, Children’s Hospital, Zhejiang University School of Medicine, Hangzhou, 310051 China; 2 National Clinical Research Center for Child Health, Children’s Hospital, Zhejiang University School of Medicine, Hangzhou, China

**Keywords:** NAFLD, Obesity, Gender differences, Sex hormones, Testosterone, Estradiol, Luteinizing hormone, Prolactin, Puberty, Fat distribution

## Abstract

**Objective:**

Previous studies have reported sex differences in non-alcoholic fatty liver disease (NAFLD) among adults; however, little is known about its occurrence in children and adolescents. This study aims to examine the prevalence of NAFLD among them and investigate the relationship between sex hormones and NAFLD.

**Method:**

This study included 2999 obese Chinese children aged 2–18 years. We examined the prevalence of NAFLD by sex, age, and Tanner stage. The regression model and principal component analysis were used to analyze the relationship between sex hormones and NAFLD.

**Results:**

The prevalence of NAFLD increased with age in both sexes, and the gender difference appeared before puberty. The prevalence in boys tended to stabilize at the age of 11 years, whereas girls reached their peak temporarily. NAFLD prevalence was positively associated with estradiol in boys (*p* = 0.011), but the opposite trend was observed in girls (*p* = 0.031). Testosterone levels decreased with the increase of NAFLD prevalence in boys (*p* < 0.001). Luteinizing hormone and prolactin were inversely associated with NAFLD prevalence in boys and girls, respectively. Results from the principal component analysis showed that sex hormone levels and fat distribution were important risk factors for the prevalence of NAFLD in obese children (*p* < 0.001).

**Conclusion:**

The significant difference in NAFLD prevalence between genders in obese children begins in early childhood. This distinction emerges long before puberty onset and tends to stabilize during late adolescence. Sex hormones are associated with NAFLD prevalence and are influenced by the Tanner stages and fat distribution.

**Supplementary Information:**

The online version contains supplementary material available at 10.1007/s12072-023-10596-9.

## Introduction

NAFLD is characterized by the excess accumulation of fat in hepatocytes, occurring in the absence of secondary causes such as excessive alcohol consumption, use of steatogenic medications, or hereditary disorders [1]. NAFLD is associated not only with morbidity and mortality from liver-related diseases but also with an elevated risk of cardiovascular diseases, type 2 diabetes, and adult mortality [2]. Given the lack of effective treatments for NAFLD and its potential impact on public health, it is essential to further understand its pathogenesis.

The global prevalence of NAFLD is experiencing a significant increase, with its onset occurring at younger ages in recent years [3]. The prevalence of childhood NAFLD has risen to 5.5% in Asia, affecting 50% of obese children [4]. Interestingly, the majority of epidemiological studies have indicated a higher prevalence among males [5]. However, no studies have yet explored NAFLD prevalence based on sex, age, or Tanner stage in children and adolescents. Studies in adults examining differences in NAFLD prevalence based on menopausal status, use of synthetic hormones, puberty onset, and conditions causing disrupted sex hormone levels, such as polycystic ovary syndrome (PCOS), strongly suggest that sex hormones may play a role in the development of NAFLD [5–7]. Sex hormones undergo significant changes during the course of puberty, similar to the hormonal changes observed during menopause [8]. While previous studies have reported that the pathogenesis of NAFLD in children may differ from that in adults [9], the relationship between sex hormones and NAFLD prevalence still requires further research.

To comprehend when and how sexual differences emerge, we calculated NAFLD prevalence based on sex, age, and Tanner stages in this study. Simultaneously, our aim was to investigate the association between NAFLD and circulating levels of sex hormones, including testosterone, estradiol, luteinizing hormone, and follicle-stimulating hormone, in both sexes. In this study, we hypothesized that the prevalence of NAFLD increases with age and exhibits significant variations at different stages of puberty. Additionally, we intend to confirm the specific correlation between sex hormones and NAFLD.

## Materials and methods

### Study population

Participants in this study were moderately or severely obese children aged 2–18 years, recruited from the Department of Endocrinology, Children’s Hospital, Zhejiang University School of Medicine between January 2003 and May 2021. Patients were included in the study if they were diagnosed with obesity, defined as having a body mass index (BMI; weight (kilograms)/height (meters)^2^) greater than the 95th percentile according to WHO references [10]. Children with elevated levels of alanine aminotransferase (ALT), aspartate aminotransferase (AST), or γ-glutamyltransferase (γGT) underwent testing for hepatitis B and C; those with negative test results were included in the study. Participants with known reasons for chronic liver diseases after clinical evaluation were excluded. Individuals exposed to specific drugs, including antidiabetic, antihyperlipidemic, antihypertensive, and hepatotoxic drugs, as well as those consuming ethanol, experiencing systemic or organic diseases, or missing essential variables, were excluded from the study. Ultimately, a total of 2999 children were included in the investigation. All participants underwent ultrasonography screening for fatty liver.

### Measurement of NAFLD features

Liver B-mode ultrasonography was performed by experienced sonographers using a Philips HP Sonos 5500 (Philips, Andover, United States). Scans were conducted after a fasting period of at least 7 h.

### Laboratory data measurements

Baseline blood samples were collected at 8:00 in the morning after a 12-h fasting period. Subsequently, all participants underwent a standard oral glucose tolerance test (OGTT). Blood glucose and insulin levels were measured at 30-min intervals over a period of 0–120 min.

Serum sex hormones, including estradiol, prolactin (PRL), follicle-stimulating hormone (FSH), luteinizing hormone (LH), and testosterone (T), were quantified using an ELISA kit (Abbott, Longford, Ireland) and analyzed using an IMMULITE 2000 XPi analyzer. Biochemistry tests were conducted on serum samples for total triglycerides (TG), cholesterol (TC), low-density lipoprotein cholesterol (LDL-C), high-density lipoprotein cholesterol (HDL-C), uric acid, as well as liver enzymes ALT, AST, and γGT. These tests were performed using an AU5800 series Analyzer (Beckman Coulter, USA).

### Anthropometric data measurements

A structured bedside interview was conducted by trained pediatric endocrinologists to gather demographic data from the study participants. The weight and height of the participants were measured while they wore lightweight clothing, excluding shoes, and were recorded to the nearest 0.1 kg and 0.1 cm, respectively. BMI was calculated as weight (kilograms)/height(meters)^2^, and its percentile was determined following the WHO standards. Age- and sex-specific BMI-z scores were then calculated based on these percentiles.

The participants' puberty stages were assessed by experienced endocrinologists using the Tanner scale. They were categorized into three stages: pre-pubertal (Tanner 1), pubertal (Tanner 2–4), and post-pubertal (Tanner 5).

Blood pressure was measured with the participants seated, and three readings were taken after a 10-min rest. The average of the last two measurements was calculated. Waist circumference was measured between the lowest costal margin and the top of the iliac crest while the participant was standing. Hip circumference was measured at the widest part of the trochanter on a horizontal level. The waist-to-hip ratio was calculated as the ratio of waist circumference to hip circumference.

### Statistical analyses

We tabulated demographic and laboratory characteristics separately for girls and boys. Continuous variables were presented as means ± SD for normally distributed data, and as the median and interquartile range for non-normally distributed data. Categorical variables were presented as counts and percentages. Significance of differences between the sexes was assessed using the *t*-test for normally distributed variables, the Kruskal–Wallis rank sum test for non-normally distributed variables, and the *χ*^2^ test for categorical variables.

As the sex hormones were non-normally distributed variables, we normalized them by using a log-transformation to avoid assuming normality in linear regression. We considered log-transformed sex hormones as independent variables and the features of fatty liver as dependent variables. Potential covariates, including age, BMI-SDS, and Tanner stages, were considered in the adjusted model. For each type of sex hormone, a specific regression model with the prevalence of fatty liver diseases as a continuous term was conducted. Additionally, we constructed a Logistic regression model for the five types of sex hormones, which were categorized into four quartiles.

The relationships between variables associated with the prevalence of NAFLD were investigated using principal component factor analysis. The number of factors was determined by the criterion of eigenvalues greater than 1. The factor patterns were illustrated with the facilitation of orthogonal and orthotran rotations, presenting as an oblique solution reference structure matrix. Small correlations were indicated by factor loadings ranging from 0.3 to 0.5, while strong correlations were indicated by factor loadings above 0.5. Multivariable logistic regression was employed to assess the associations of sex hormones with the risk of hepatic steatosis. The results are presented as estimated regression coefficients, odds ratios with their corresponding 95% confidence intervals, and *p* values from the likelihood ratio test.

In all the statistical analyses, *p* values < 0.05 were considered statistically significant. Statistical analyses and graph plotting were performed using the R software program, version 4.0 (R Foundation for Statistical Computing).

## Results

### Prevalence of NAFLD

**Fig. 1:**
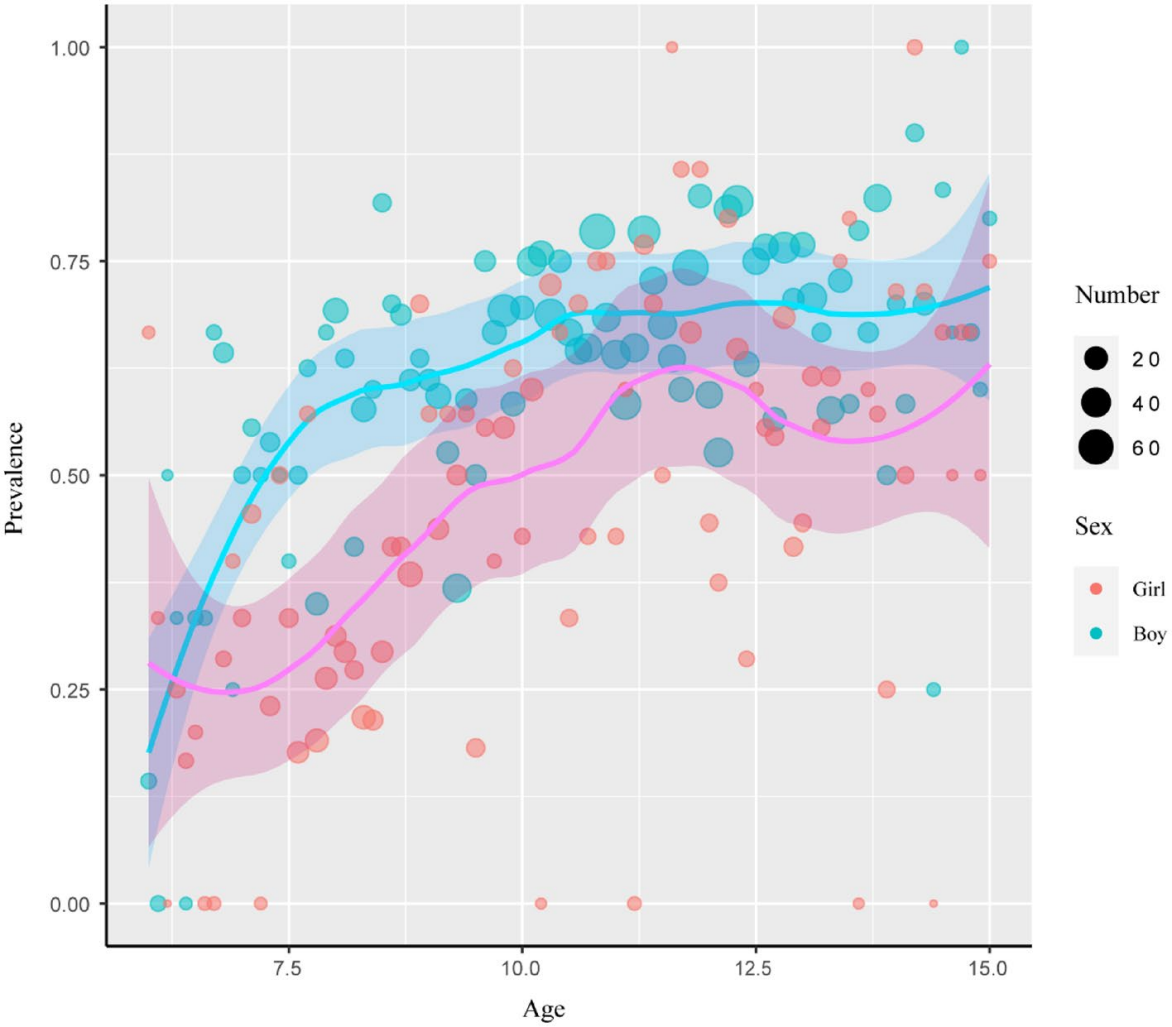
Age- and sex-specific prevalence of fatty liver in obese children between 6 and 15 years. Solid lines are prevalence estimates with shaded areas indicating 95% Cis. Each circle represents a sample with specific age and sex. For an accurate estimate, the children younger than 6 years and older than 15 years were removed when drawing the plot due to their fewer numbers

**Fig. 2:**
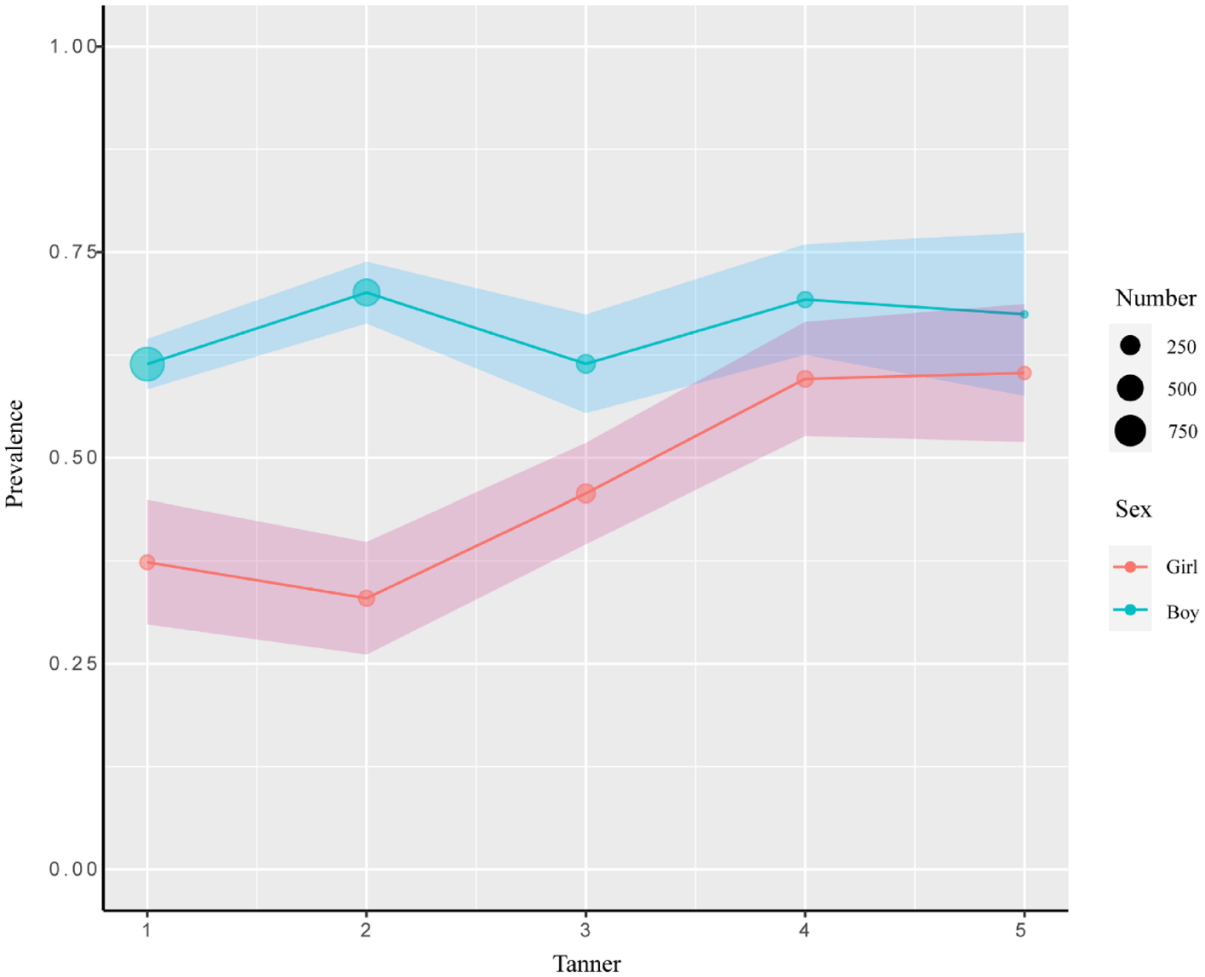
Tanner- and sex-specific prevalence of fatty liver disease in obese children. Solid lines are prevalence rate changed with tanner stages. Shaded areas indicate 95% Cis. Each circle represents a sample of specific tanner stages and sex

The rate of hepatic steatosis diagnosed by ultrasound in obese children differs with age and sex. Among boys, the initial prevalence rate was around 30% at the age of 2–4 years. It decreased to 25% at the age of 5 years, increased to 71% at the age of 10 years, and then stabilized (Supplementary Table 1, Fig. 1). Among girls, the prevalence rate was 15% at the age of 5 years, increased to 65% at the age of 11 years, and then showed variation until the age of 15 years (Supplementary Table 1, Fig. 1). The Tanner-specific pattern reveals that the prevalence rate in girls rises in parallel with the Tanner stages; however, the prevalence rate in boys fluctuated and reached the peak at Tanner stages 2 and 4(Fig. 2). Overall, the prevalence among boys was significantly higher than that among girls, and both genders exhibited distinct patterns related to pubertal stages and development.

### Anthropometric and metabolic characteristics

The characteristics of the children are listed in Table 1. The mean age for all children was 10.61 years (SD: 2.50). The average age of boys and girls in our study was 10.82 years (SD: 2.37) and 10.12 years (SD: 2.72), respectively. Overall, 256 (8.54%) children were younger than 7 years old, 2540 (84.69%) children were between 7 and 14 years old, and 203 (6.77%) children were older than 14 years old. The average BMI Z-score was 3.26 (SD: 1.15)–3.41 (SD: 1.22) for boys and 2.92 (SD: 0.89) for girls. The mean waist circumference was 89.40 cm (SD: 11.38)–91.40 cm for boys (SD: 10.55) and 84.84 cm (SD: 11.88) for girls. In total, 1142 (38.08%) children were pre-pubertal, 1640 (54.68%) were pubertal, and 217 (7.24%) were post-pubertal. Among boys, 984 (47.28%) children were pre-pubertal, 1011 (48.58%) were pubertal, and 86 (4.13%) were post-pubertal. Among girls, 158 (17.21%) were pre-pubertal, 629 (68.52%) were pubertal, and 131 (14.27%) were post-pubertal. Most of the characteristics were significantly different by sex, although some metrics related to glucose metabolism were similar. Boys’ metrics were higher in terms of biochemical analyses and testosterone levels, whereas other sex hormones were higher in girls.

**Table 1:** Demographic characteristics, sex hormones distributions, pubertal stages, and biochemical tests in all the children from our study by sex (*N* = 2999)

Characteristic	Overall	Boys	Girls	*p*
	*N* = 2999	*N* = 2081	*N* = 918	
Demographic characteristics	Mean (SD)	Mean (SD)	Mean (SD)	
Age (years)	10.61 (2.50)	10.82 (2.37)	10.12 (2.72)	< 0.001
Height (cm)	148.27 (14.66)	149.99 (14.38)	144.36 (14.55)	< 0.001
Weight (kg)	63.56 (18.64)	65.65 (17.89)	58.82 (19.45)	< 0.001
BMI (kg/m^2^)	28.26 (4.16)	28.62 (3.84)	27.43 (4.71)	< 0.001
BMI Z-score	3.26 (1.15)	3.41 (1.22)	2.92 (0.89)	< 0.001
Waist circumference (cm)	89.40 (11.38)	91.40 (10.55)	84.84 (11.88)	< 0.001
Hip circumference (cm)	94.86 (11.25)	95.60 (10.42)	93.17 (12.78)	< 0.001
Waist/Hip circumference	0.93 (0.09)	0.95 (0.09)	0.91 (0.08)	< 0.001
SBP (mmHg)	120.62 (14.12)	121.62 (14.12)	118.34 (13.87)	< 0.001
DBP (mmHg)	70.05 (9.53)	70.32 (9.38)	69.46 (9.83)	0.009

Tanner stages	*n* (%)	*n* (%)	*n* (%)	
1, pre-puberty	1142 (38.08%)	984 (47.28%)	158 (17.21%)	< 0.001
2–4, puberty	1640 (54.68%)	1011 (48.58%)	629 (68.52%)	
5, mature	217 (7.24%)	86 (4.13%)	131 (14.27%)	

Sex hormones	Median (IQR)	Median (IQR)	Median (IQR)	
Estradiol (pmol/L)	88.08 (73.40–130.61)	88.05 (73.40–115.00)	109.92 (73.40–167.72)	< 0.001
FSH (U/L)	2.10 (0.90–3.90)	2.10 (0.87–3.62)	2.30 (1.10–4.60)	< 0.001
LH (U/L)	0.32 (0.10–2.10)	0.32 (0.10–1.83)	0.30 (0.10–3.81)	0.007
PRL (mU/L)	177.23 (127.54–254.00)	177.23 (122.45–235.00)	207.02 (144.58–293.12)	< 0.001
T (nmol/L)	0.693 (0.693–1.640)	0.693 (0.693–2.290)	0.693 (0.693–1.150)	< 0.001

Biochemical tests	Mean (SD)	Mean (SD)	Mean (SD)	
ALT (U/L)	47.09 (48.25)	52.71 (52.91)	34.36 (32.05)	< 0.001
AST (U/L)	35.21 (26.14)	37.65 (28.09)	29.69 (20.01)	< 0.001
UA (umol/L)	390.40 (96.76)	397.78 (99.27)	373.66 (88.60)	< 0.001
TG (mmol/L)	1.37 (0.81)	1.36 (0.82)	1.39(0.79)	0.15
TC (mmol/L)	4.30(0.86)	4.34 (0.84)	4.21 (0.90)	< 0.001
HDL (mmol/L)	1.22 (0.27)	1.23 (0.27)	1.19 (0.26)	< 0.001
LDL (mmol/L)	2.58 (0.66)	2.59 (0.65)	2.56 (0.68)	0.02
OGTT 0′Glucose (mmol/L)	5.36 (0.62)	5.37 (0.60)	5.34 (0.65)	0.09
OGTT 30′Glucose (mmol/L)	8.18 (1.43)	8.23 (1.39)	8.06 (1.50)	< 0.001
OGTT 120′Glucose (mmol/L)	6.84 (1.47)	6.79 (1.42)	6.93 (1.58)	0.07
OGTT 0′Insulin (mmol/L)	37.81 (74.26)	38.13 (75.46)	37.09 (71.51)	0.96
OGTT 30′Insulin (mmol/L)	261.30 (566.84)	277.87 (606.43)	223.76 (462.86)	0.02
OGTT 120′Insulin (mmol/L)	170.62 (434.99)	174.53 (437.21)	161.78 (430.02)	0.19

BMI, body mass index; SBP, systolic blood pressure; DBP, diastolic blood pressure; ALT, alanine aminotransferase; AST, aspartate aminotransferase; FSH, follicle-stimulating hormone; LH, luteinizing hormone; PRL, prolactin; T, testosterone; UA, uric acid; TG, triglycerides; TC, cholesterol; OGTT, oral glucose tolerance test

### Sex hormone relations with fatty liver

We constructed unadjusted and adjusted regression models to evaluate the independent impact of sex hormones on NAFLD prevalence (refer to Tables 2 and 3). Odds ratios (ORs) and 95% confidence intervals (CIs) are presented in Tables 2 and 3. The ORs for estradiol remained significant between the unadjusted and adjusted models for both girls and boys (P < 0.05). The OR (95% CI) for NAFLD was 0.97 (95% CI: 0.83, 1.11) in girls and 1.02 (95% CI: 0.93, 1.11) in boys with a per interquartile increase of estradiol in adjusted model. PRL was inversely associated with the risk of NAFLD in girls, with an OR of 0.80 (95% CI: 0.70–0.91). No associations were observed for FSH, LH and testosterone in girls. Boys in the lowest LH quartile had an increased risk of NAFLD prevalence compared to the highest quartile (OR: 0.84, 95% CI: 0.85–1.00). The OR (95% CI) for NAFLD was 0.99 (95% CI: 0.89, 1.11) in boys with a per interquartile increase of testosterone in adjusted model (Table 3).

**Table 2:** Associations between sex hormones and NAFLD prevalence in girl population

Exposure	Cases/Person	Unadjusted model [OR (95%CI)]	Adjusted model [OR (95%CI)]
Estradiol		1.31 (1.17–1.47)	0.97 (0.84–1.11)
Q1	94/257	1 (Ref)	1 (Ref)
Q2	80/202	1.10 (0.76–1.60)	0.95 (0.64–1.42)
Q3	133/228	2.21 (1.43–3.43)	1.41 (0.94–2.10)
Q4	122/231	2.93(1.95–4.39)	0.77 (0.49–1.19)
*p* for trend		< 0.001	0.031
FSH		1.34 (1.19–1.51)	0.96 (0.83–1.13)
Q1	90/223	1 (Ref)	1 (Ref)
Q2	86/235	0.99 (0.69–1.43)	0.74 (0.49–1.11)
Q3	114/227	1.15 (0.80–1.66)	0.91 (0.59–1.41)
Q4	139/233	0.66 (0.45–0.96)	0.84 (0.52–1.35)
*p* for trend		< 0.001	0.497
LH		1.49 (1.31–1.71)	0.87 (0.71–1.06)
Q1	55/136	1 (Ref)	1 (Ref)
Q2	114/319	0.82 (0.54–1.24)	0.79 (0.52–1.22)
Q3	112/233	1.36 (0.89–2.09)	0.64 (0.39–1.06)
Q4	148/230	2.66 (1.72–4.11)	0.68 (0.37–1.24)
*p* for trend		< 0.001	0.393
PRL		0.90 (0.80–1.01)	0.80 (0.70–0.91)
Q1	111/229	1 (Ref)	1 (Ref)
Q2	111/230	0.99 (0.69–1.43)	0.87 (0.59–1.29)
Q3	119/229	1.15 (0.80–1.66)	0.87 (0.58–1.30)
Q4	88/230	0.66 (0.45–0.96)	0.47 (0.32–0.71)
*p* for trend		0.023	0.002
T		1.51 (1.33–1.71)	1.08 (0.92–1.26)
Q1	64/178	1( Ref)	1 (Ref)
Q2	135/353	1.10 (0.76–1.60)	1.12 (0.75–1.66)
Q3	87/157	2.21 (1.43–3.43)	1.49 (0.93–2.38)
Q4	143/230	2.93 (1.95–4.39)	1.18 (0.73–1.91)
*p* for trend		< 0.001	0.407

Adjusted model included age, BMI-SDS and tanner stages as co-variants

FSH, follicle-stimulating hormone; LH, luteinizing hormone; PRL, prolactin; T, testosterone

**Table 3:** Associations between sex hormones and NAFLD prevalence in boy population

Exposure	Cases/Person	Unadjusted model [OR (95%CI)]	Adjusted model [OR (95%CI)]
Estradiol		1.10 (1.01–1.20)	1.02 (0.93–1.11)
Q1	267/461	1 (Ref)	1 (Ref)
Q2	383/580	1.42 (1.10–1.83)	1.38 (1.06–1.80)
Q3	361/522	1.64 (1.26–2.13)	1.44 (1.09–1.89)
Q4	336/521	1.33 (1.03–1.72)	1.06 (0.80–1.39)
*p* for trend		0.002	0.011
FSH		1.09 (1.01–1.18)	0.88 (0.79–0.98)
Q1	314/519	1 (Ref)	1 (Ref)
Q2	334/515	1.21 (0.94–1.55)	0.97 (0.73–1.27)
Q3	355/527	1.34 (1.04–1.73)	0.80 (0.59–1.09)
Q4	344/520	1.28 (0.99–1.65)	0.70 (0.51–0.97)
*p* for trend		0.106	0.104
LH		1.13 (1.03–1.24)	0.84 (0.74–0.96)
Q1	161/262	1 (Ref)	1 (Ref)
Q2	485/778	1.04 (0.78–1.39)	0.98 (0.73–1.33)
Q3	343/520	1.22 (0.89–1.65)	0.76 (0.53–1.08)
Q4	358/521	1.38 (1.01–1.88)	0.62 (0.42–0.93)
*p* for trend		0.068	0.047
PRL		0.93 (0.86–1.01)	0.92 (0.85–1.00)
Q1	350/520	1 (Ref)	1 (Ref)
Q2	347/520	0.97 (0.75–1.26)	0.98 (0.75–1.28)
Q3	318/520	0.77 (0.59–0.99)	0.73 (0.56–0.96)
Q4	332/521	0.85 (0.66–1.10)	0.84 (0.65–1.10)
*p* for trend		0.135	0.074
T		1.17 (1.07–1.28)	0.99 (0.89–1.11)
Q1	196/365	1 (Ref)	1 (Ref)
Q2	499/755	1.68 (1.30–2.17)	1.72 (1.32–2.24)
Q3	302/438	1.93 (1.45–2.58)	1.51 (1.11–2.04)
Q4	350/523	1.73 (1.31–2.28)	0.89 (0.62–1.27)
*p* for trend		< 0.001	< 0.001

Adjusted model included age, BMI-SDS and tanner stages as co-variants

FSH, follicle-stimulating hormone; LH, luteinizing hormone; PRL, prolactin; T, testosterone

### Principal component analysis

Principal component analysis extracted three factors from 17 variables, including demographic characteristics, biochemical tests, sex hormones, and metrics of glucose metabolism. In both sexes, factor loadings indicated close associations among uric acid, estradiol, LH, testosterone, waist circumference, and hip circumference, which can be summarized as 'sex hormone and fat distribution'. The second factor consisted of ALT, triglycerides, cholesterol, the triglyceride glucose index (TyG), and LDL, which can be regarded as related to lipid metabolism. The third factor is classified as 'glucose metabolism', formed by glucose, homeostatic model assessment for insulin resistance (HOMA-IR), and the insulin sensitivity index (ISI) (see Supplementary Table 2).

### Multiple logistic regression analysis

To reveal the potential role of the components as predictors of NAFLD prevalence in obese children, a stepwise multiple logistic regression model was constructed separately for boys and girls. The dependent variable was the existence of fatty liver detected by ultrasonography, while the independent variables consisted of three principal components. Age, BMI Z-score, and pubertal stage were set as adjusting variables. The factors of sex hormones and fat distribution were significantly associated with the prevalence of fatty liver in both boys and girls, and this association was greatly affected by Tanner stages, BMI Z-score, and age (see Table 4).

**Table 4:** Multivariate logistic regression analysis with fatty liver disease as the dependent variable and the principal component analysis derived factors as the independent variable in boys (*n* = 2081) and girls (*n* = 918)

Principal component	Boys			Girls		
	OR	95%CI	*p*	OR	95%CI	*p*
Sex hormones and fat distribution	1.31	1.09–1.57	< 0.001	1.40	1.05–1.87	0.02
Lipid metabolism	1.26	1.13–1.39	< 0.001	1.60	1.37–1.88	< 0.001
Glucose metabolism	1.08	0.98–1.20	n.s.	1.39	1.18–1.64	< 0.001
Adjusting variables						
BMI Z-score	1.34	1.17–1.54	< 0.001	1.52	1.22–1.91	< 0.001
Age	1.28	1.16–1.42	< 0.001	1.24	1.11–1.40	< 0.001
Tanner stages						
Prepubertal stage	3.35	1.88–5.92	< 0.001	1.71	0.90–3.24	n.s.
Intra-pubertal stage	2.35	1.38–3.93	< 0.001	1.65	1.02–2.67	0.04

Coef, regression coefficient; OR, odds ratio; CI, confidence interval; n.s., not significant

## Discussion

In this large sample population, our findings reinforce the observation that boys have a higher prevalence of NAFLD compared to girls, regardless of age and Tanner stage. The gender difference is apparent in pre-puberty and narrows in late puberty. For girls, estradiol and PRL were inversely associated with the prevalence of NAFLD, while in boys, estradiol was positively related to NAFLD prevalence, whereas LH and testosterone had a negative correlation. Principal component analysis identified three important components representing the potential impact of markers of sex hormones and fat distribution, lipid metabolism, and glucose metabolism. Logistic regression analysis reveals the importance of sex hormones and fat distribution as factors associated with the prevalence of fatty liver in both sexes, and puberty may increase the risk of fatty liver.

Although the susceptibility to pediatric NAFLD in boys, as found in our study cohort, has been demonstrated in previous studies [4, 11, 12], our study still provided some new and interesting findings. As a supplement to the results discovered in a meta-analysis based on Asian children, NAFLD is becoming prevalent among obese children, especially boys, after the age of 10 [4]. Furthermore, our study revealed a rapid increase in NAFLD prevalence in children after the age of 6, coinciding with the emergence of sexual differences. Meanwhile, the Healthy Start Study of 286 children aged 4–8 years, which investigated liver fat composition by MRI-H1 in children before pubertal onset, reported that hepatic steatosis might begin in early childhood. They found a positive association between fasting glucose, triglycerides, and liver fat deposition, but only in boys [13]. Together with our results, these findings provide stronger evidence and clinical significance that young boys are more susceptible to NAFLD.

Notably, our study revealed that the prevalence of fatty liver in obese preschool children reached 21–28%, which is much higher than we expected. Severe obesity might be one reason for this high prevalence. It also underscores the importance of genetic metabolic screening for young children with NAFLD since many rare, inherited metabolic disorders can also cause fatty liver in infants and children [14]. Additionally, the gender difference became apparent at around 6 years of age when the sex hormone axis remains inactive. Interestingly, focal development of the zona reticularis (ZR) of the human adrenal cortex begins at around 5 years of age, coinciding with the onset of increased levels of dehydroepiandrosterone (DHEA) and dehydroepiandrosterone sulfate (DHEAS) [15]. We speculate that, rather than testosterone and estradiol, other hormones like DHEA and DHEAS, which are related to the sex of the subject, may influence the prevalence of NAFLD. Some studies have reported a negative association between DHEAS and the severity of NAFLD in both teenagers and adults [16, 17]. Further studies are needed to investigate the relationship between adrenal androgens and NAFLD in pre-pubertal children. Meanwhile, central obesity, insulin resistance, and weight gain are major risk factors for NAFLD [18]. Additionally, other factors related to sex, such as sex-chromosome complement, genetic and epigenetic factors, circadian rhythm, gut microbiota, individual lifestyle, cultural sex bias in parental feeding practices, and societal ideals of body size, might also play a role in the sexual difference in pre-pubertal NAFLD prevalence [5, 19, 20]. Another interesting finding was the peak in the prevalence rate in girls at around 11 years of age. Insulin resistance increased significantly during the pubertal transition, peaked at Tanner stage 3, and returned to pre-pubertal levels at Tanner stage 5 in girls [12, 21]. Insulin resistance causes NAFLD by increasing de novo lipogenesis and increasing FFA flux to the liver [22], which might explain the fluctuation of NAFLD prevalence during the pubertal transition in girls.

Our results regarding the association between sex hormones and NAFLD prevalence were consistent with previous findings in human and animal models [17, 23, 24]. In contrast to males, estradiol serves as a protective factor against liver disease in female mice. Hepatic steatosis is linked to a slight reduction in circulating testosterone levels in male mice [23]. Testosterone levels were positively correlated with improvements in steatosis and fibrosis in boys but displayed an inverse association in girls. Furthermore, higher estradiol levels were positively linked to the severity of hepatic ballooning in both sexes [17]. Estrogen signaling in both sexes is intertwined with lipid metabolism in the liver [19]. Under physiological conditions, it prevents hepatic fat accumulation in females by inhibiting the expression of genes involved in de novo lipogenesis [19]. Furthermore, androgen signaling also regulates hepatic lipid metabolism. In males, the androgen signaling pathway suppresses the genes involved in DNL and lipid storage by up-regulating PPARα (peroxisome proliferator-activated receptor α) signaling to promote fatty acid oxidation; however, it disrupts hepatic lipid metabolism in female mice [19]. These findings can help explain the divergent associations between different sex hormones and NAFLD. Zhang et al. [25], reported that hepatic lipid accumulation can be improved by PRL/prolactin receptor via the CD36 pathway. Another study by Shao et al. [26] found that hepatic TG accumulation could be reduced in female mice, and liver steatosis could be alleviated in male mice when given PRL in a high-fat diet-induced NAFLD model. Considering that a decrease in PRL has been observed in NAFLD patients of both genders [24, 27], whether there are sexual differences between serum PRL and NAFLD remains unknown.

During this study, we observed a weak correlation between sex hormones and ALT (Supplementary Table 3). There was a strong association between NAFLD and parameters related to body fat distribution and sex hormones. Denzer et al. [11] also arrived at the same conclusion as the one we stated earlier. Waist and hip circumferences serve as indicators of body fat distribution. Gender dimorphism in fat tissue across various body areas is influenced by sex hormones through depot- and time-specific estrogen receptor expression, the active–passive transition of lipid metabolism-related proteins, circulating adipokines, immune response, and more [28, 29]. Males tend to accumulate abdominal and visceral fats, such as Android fat, which are risk factors for NAFLD, while females store white adipose tissues in the subcutaneous and femoral regions, like Gynoid fats [29, 30]. A previous study by our group reported that the Android/Gynoid ratio in pre-pubertal boys, an index for gender-specific abdominal fat distribution, increases before puberty onset [30]. In addition to the effects of sex hormones, differential fat distribution partially explains the early increase in NAFLD prevalence in pre-pubertal boys.

Despite the large sample size of obese children and adolescents with comprehensive clinical examinations of obesity complications, our research has several limitations. Since the study was cross-sectional, it could not dynamically observe the disease progression in different pubertal stages. Furthermore, the sex hormone levels were very low before puberty, with some falling below the detection limits of the assays, which might have introduced certain statistical errors. However, the low sex hormone levels emphasize the intriguing sexual dimorphism of NAFLD before puberty. Additionally, this study population predominantly consisted of Chinese children with moderate to severe obesity who were hospitalized, with the number of boys nearly double that of girls. These factors might lead to selection bias and an under-representation of other racial groups and the general population.

In conclusion, the prevalence of NAFLD is higher in obese children in China, and the sex difference becomes apparent in early childhood, at around 6 years of age, long before sex hormones start to rise. Liver function and liver imaging examinations might be necessary for such young children, especially those who are obese. Sex hormones are related to the severity of NAFLD, and they are influenced by puberty and abdominal fat accumulation. While our study has provided new findings regarding sexual differences in obese children, there are still unanswered questions, such as the sex-specific prevalence of NAFLD in young children, the gender-related effects of NAFLD beyond hormones, and the mechanism of NAFLD sexual dimorphism in kids.

### Supplementary Information

Below is the link to the electronic supplementary material.

Supplementary file1 (DOCX 34 kb)

## Data Availability

The datasets generated and analyzed during the current study are not public but are available from the corresponding author upon reasonable request.
