# Supplementary material for: Gender differences in non-alcoholic fatty liver disease in obese children and adolescents: a large cross-sectional study
Source: Hepatol Int. 2023 Oct 20;18(1):179–87. doi: 10.1007/s12072-023-10596-9 (PMC10858100; doi:10.1007/s12072-023-10596-9)
Supplement: Supplementary file 1 — Supplementary file1 (DOCX 34 kb) [file 12072_2023_10596_MOESM1_ESM.docx]

**Supplementary Table 1** Age- and sex-specific prevalence of fatty liver in obese children between 2-18 years.

| **age** | **all** | | | **boy** | | | **girl** | | | |
| --- | --- | --- | --- | --- | --- | --- | --- | --- | --- | --- |
|  | n | prevalence | CI | n | prevalence | CI | n | prevalence | CI |  |
| **2-4** | 17 | 0.28 | 0.17-0.40 | 13 | 0.30 | 0.17-0.44 | 4 | 0.24 | 0.03-0.44 |  |
| **5** | 18 | 0.21 | 0.13-0.30 | 13 | 0.25 | 0.14-0.37 | 5 | 0.15 | 0.03-0.27 |  |
| **6** | 36 | 0.32 | 0.24-0.41 | 24 | 0.41 | 0.28-0.53 | 12 | 0.23 | 0.12-0.35 |  |
| **7** | 95 | 0.41 | 0.34-0.47 | 58 | 0.54 | 0.45-0.64 | 37 | 0.29 | 0.21-0.37 |  |
| **8** | 141 | 0.48 | 0.43-0.54 | 89 | 0.62 | 0.54-0.70 | 52 | 0.35 | 0.27-0.43 |  |
| **9** | 201 | 0.56 | 0.51-0.61 | 152 | 0.59 | 0.53-0.65 | 49 | 0.48 | 0.38-0.58 |  |
| **10** | 328 | 0.68 | 0.64-0.73 | 268 | 0.71 | 0.66-0.75 | 60 | 0.61 | 0.51-0.70 |  |
| **11** | 317 | 0.68 | 0.64-0.72 | 264 | 0.69 | 0.64-0.73 | 53 | 0.65 | 0.55-0.76 |  |
| **12** | 296 | 0.68 | 0.64-0.73 | 236 | 0.72 | 0.67-0.77 | 60 | 0.56 | 0.47-0.65 |  |
| **13** | 183 | 0.66 | 0.60-0.71 | 141 | 0.69 | 0.62-0.75 | 42 | 0.58 | 0.46-0.69 |  |
| **14** | 85 | 0.69 | 0.61-0.77 | 55 | 0.71 | 0.60-0.81 | 30 | 0.67 | 0.53-0.80 |  |
| **15-18** | 59 | 0.74 | 0.64-0.83 | 34 | 0.72 | 0.60-0.85 | 25 | 0.76 | 0.61-0.90 |  |

n stand for the NAFLD patient number; CI, confidence interval;

**Supplementary Table 2 Oblique solution reference structure of matrix of principal component analysis performed with potential risk factors for NAFLD in the total 2999 children.**

| **Variable** | **Factors** | | |
| --- | --- | --- | --- |
|  | **Sex hormone and** **fat distribution** | **Lipid metabolism** | **Glucose metabolism** |
| **Boys** |  |  |  |
| ALT | 0.121 | **0.274** | 0.127 |
| Uric acid | **0.626** | 0.117 | 0.154 |
| Triglycerides | 0.013 | **0.784** | 0.187 |
| Cholesterol | -0.063 | **0.819** | -0.158 |
| Estradiol | **0.378** | -0.009 | -0.109 |
| FSH | 0.173 | 0.036 | -0.027 |
| LH | **0.722** | -0.015 | 0.063 |
| T | **0.736** | -0.103 | 0.033 |
| Glucose | -0.111 | 0.225 | **0.607** |
| OGTT 120'Glucose | 0.002 | 0.281 | **0.566** |
| HOMA-IR | 0.164 | -0.06 | **0.714** |
| TyG | 0.037 | **0.791** | 0.268 |
| ISI | -0.262 | 0.015 | **-0.743** |
| PRL | 0.048 | 0.069 | -0.093 |
| LDL | 0.045 | **0.804** | -0.124 |
| Waist circumference | **0.778** | 0.101 | 0.131 |
| Hip circumference | **0.846** | 0.064 | 0.161 |
| **Girls** |  |  |  |
| ALT | 0.221 | **0.341** | 0.254 |
| Uric acid | **0.411** | 0.232 | 0.217 |
| Triglycerides | -0.006 | **0.755** | 0.217 |
| Cholesterol | 0.025 | **0.805** | -0.133 |
| Estradiol | **0.618** | -0.073 | -0.05 |
| FSH | **0.273** | 0.034 | 0.22 |
| LH | **0.685** | -0.018 | 0.042 |
| T | **0.598** | 0.024 | 0.093 |
| Glucose | -0.113 | 0.098 | **0.554** |
| OGTT 120'Glucose | 0.129 | 0.219 | **0.535** |
| HOMA-IR | 0.092 | -0.07 | **0.753** |
| TyG | -0.006 | **0.753** | **0.357** |
| ISI | -0.181 | -0.011 | **-0.763** |
| PRL | 0.169 | 0.04 | -0.086 |
| LDL | 0.076 | **0.819** | -0.12 |
| Waist circumference | **0.814** | 0.114 | 0.147 |
| Hip circumference | **0.857** | 0.03 | 0.177 |

Factors loading indicating small (>0.3) or strong correlations (>0.5) are shown in bold. Abbreviations: ALT, Alanine aminotransferase; FSH, Follicle-stimulating hormone; LH: Luteinizing hormone; PRL: prolactin; T: Testosterone; OGTT: Oral glucose tolerance test; HOMA-IR: homeostasis model assessment of insulin resistance; TyG: triglyceride glucose index; ISI: insulin sensitivity index.

**Supplementary Table 3** Linear associations between the ALT and sex hormone among boys (n=2081) and girls (n=918) with obesity.

|  |  | **ALT** | |
| --- | --- | --- | --- |
|  |  | **r** | **p** |
| **E2 (pmol/L)** | boys | 0.033 | 0.149 |
|  | girls | 0.039 | 0.204 |
| **FSH (U/L)** | boys | -0.01 | 0.423 |
|  | girls | **0.123** | **<0.001** |
| **LH (U/L)** | boys | 0.009 | 0.635 |
|  | girls | 0.083 | 0.306 |
| **T (nmol/L)** | boys | **-0.047** | **0.026** |
|  | girls | **0.147** | **<0.001** |
| **PRL (mU/L)** | boys | **-0.051** | **0.028** |
|  | girls | -0.035 | 0.288 |

Data are Spearman’s correlation coefficients(r) and respective p-values. Abbreviations: ALT, Alanine aminotransferase; E2: Estradiol; FSH, Follicle-stimulating hormone; LH: Luteinizing hormone; PRL: prolactin; T: Testosterone.
